# The age‐related changes in 40 Hz Auditory Steady‐State Response and sustained Event‐Related Fields to the same amplitude‐modulated tones in typically developing children: A magnetoencephalography study

**DOI:** 10.1002/hbm.26013

**Published:** 2022-07-14

**Authors:** Vardan Arutiunian, Giorgio Arcara, Irina Buyanova, Militina Gomozova, Olga Dragoy

**Affiliations:** ^1^ Center for Language and Brain HSE University Moscow Russia; ^2^ IRCCS San Camillo Hospital Venice Italy; ^3^ Institute of Linguistics Russian Academy of Sciences Moscow Russia

**Keywords:** 40 Hz Auditory Steady‐State Response, age‐related changes, amplitude‐modulated tones, auditory gamma oscillations, children, magnetoencephalography (MEG), sustained Event‐Related Fields

## Abstract

Recent studies have revealed that gamma‐band oscillatory and transient evoked potentials may change with age during childhood. It is hypothesized that these changes can be associated with a maturation of GABAergic neurotransmission and, subsequently, the age‐related changes of excitation–inhibition balance in the neural circuits. One of the reliable paradigms for investigating these effects in the auditory cortex is 40 Hz Auditory Steady‐State Response (ASSR), where participants are presented with the periodic auditory stimuli. It is known that such stimuli evoke two types of responses in magnetoencephalography (MEG)—40 Hz steady‐state gamma response (or 40 Hz ASSR) and auditory evoked response called sustained Event‐Related Field (ERF). Although several studies have been conducted in children, focusing on the changes of 40 Hz ASSR with age, almost nothing is known about the age‐related changes of the sustained ERF to the same periodic stimuli and their relationships with changes in the gamma strength. Using MEG, we investigated the association between 40 Hz steady‐state gamma response and sustained ERF response to the same stimuli and also their age‐related changes in the group of 30 typically developing 7‐to‐12‐year‐old children. The results revealed a tight relationship between 40 Hz ASSR and ERF, indicating that the age‐related increase in strength of 40 Hz ASSR was associated with the age‐related decrease of the amplitude of ERF. These effects were discussed in the light of the maturation of the GABAergic system and excitation–inhibition balance development, which may contribute to the changes in ASSR and ERF.

## Graphical abstract

Using magnetoencephalography (MEG), we aimed to investigate the association between 40 Hz steady‐state gamma response and sustained ERF response to the same stimuli and also their age‐related changes in the group of 30 typically developing 7‐to‐12‐year‐old children. The results revealed a tight relationship between 40 Hz ASSR and ERF.

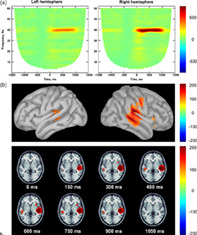

## 1 INTRODUCTION

It is hypothesized that cortical gamma‐band oscillations (30–150 Hz), which are measured with magnetoencephalography (MEG)/electroencephalography (EEG), are intimately associated with the state of the balance between excitation (E) and inhibition (I) in the neural circuits (Atallah & Scanziani, 2009; Ben‐Ari, 2014; Sohal & Rubenstein, 2019; Tang et al., 2021). Recent studies in animals using the optogenetic technique have demonstrated that the gamma‐aminobutyric acidergic (GABAergic) interneurons, expressing calcium‐binding protein parvalbumin (PV+ inhibitory neurons), play a significant role in E/I balance regulation in neural networks, being involved in many high‐order neuronal regulatory processes (Agetsuma et al., 2018; Espinoza et al., 2018; Ferguson & Gao, 2018; Magueresse & Monyer, 2013). According to existing findings, the GABA inhibitory system matures relatively late in development: it has been revealed in animals (see Williams et al., 2009; Zhang et al., 2011) and humans, using the Western blot analysis of postmortem tissue, immunocytochemistry, tissue autoradiography, and proton magnetic resonance spectroscopy (Kilb, 2012; Pinto et al., 2010; Silveri et al., 2013; Xu et al., 2011). Some authors have suggested that the GABAergic system is involved in the generation of gamma oscillations and assumed that a late maturation of GABA neurotransmission could be related to the difficulties of registering gamma oscillations and to the age‐related changes in the strength of this response in the pediatric population (Edgar et al., 2016).

There are several studies in children, using MEG/EEG, which focus on the age‐related changes in auditory gamma oscillations, using 40 Hz Auditory Steady‐State Response, or 40 Hz ASSR (Aoyagi et al., 1994; Cho et al., 2015; Edgar et al., 2016; Maurizi et al., 1990; Poulsen et al., 2009; Rojas et al., 2006; Seymour et al., 2020; Stapells et al., 1988; Stroganova et al., 2020). According to this paradigm, participants are presented with amplitude‐modulated tones or click trains at a regular gamma frequency, usually at 40 Hz (Pellegrino et al., 2019; Thut et al., 2011). The activation of neuronal populations in the primary auditory cortex (or in the vicinity of this area) becomes gradually aligned in phase with the perceiving sound and reaches a stable power increase at ~40 Hz during sound presentation. Stapells et al. (1988) in the study with typically developing infants, aged 3 weeks to 28 months, did not show a clear 40 Hz ASSR. Another study with 32 full‐term newborns and 10 children aged 5–8 years also reported the difficulties to detect 40 Hz steady‐state gamma response (Maurizi et al., 1990). However, the authors highlighted that 40 Hz ASSR became more stable, and test–retest reliability improved with age. In a large sample of participants (*N* = 188, aged 8–22 years), Cho et al. (2015) have examined evoked power and the phase‐locking factor (PLF) of 40 Hz ASSR. They found that 40 Hz evoked power as well as PLF increased from 8 to 16 years of life. The similar age‐related increase in the power of 40 Hz ASSR was shown in another study with 56 typically developing children aged 7–14 years old (Edgar et al., 2016), although, as the authors mentioned, 40 Hz ASSR was not observed in the majority of participants. In a more recent study, Stroganova et al. (2020) also have shown the monotonic increase of 40 Hz ASSR with age in a group of 7‐to‐12‐year‐old typically developing boys (*N* = 35). In the only longitudinal study, Poulsen and colleagues (2009) aimed to compare the amplitude of 40 Hz ASSR in children aged 10 and then 11.5 years of age. They demonstrated the larger 40 Hz ASSR in children aged 11.5 years of age compared to 10 year olds. No differences were found between 11.5‐year‐old children and adults. Summarizing, the previous findings have reported the age‐related increase in strength of 40 Hz auditory steady‐state gamma until early adolescence and hypothesized that the maturation of the GABAergic inhibitory system might contribute to these changes of the auditory response in the pediatric population.

Some insight into the neural mechanisms of the generation of gamma oscillations comes from the studies in animals (Bartos et al., 2007; Cardin et al., 2009; Carlén et al., 2012; Sivarao et al., 2016; Sohal et al., 2009). Sivarao et al. (2016) have suggested that the key part of the neural circuitry responsible for entrainment to exogenous stimuli involves the GABAergic basket cells (PV+ interneurons) and the pyramidal neurons of the upper layers of the auditory cortex. They have shown with 40 Hz ASSR paradigm in rats that, in particular, the occupation of *N*‐methyl‐Dd‐aspartate (NMDA) channel on PV+ cells reduced 40 Hz auditory steady‐state gamma response. This demonstrated that NMDA receptor activation on PV+ interneurons is crucial for normal 40 Hz auditory synchronization. Additionally, in the study with optogenetic manipulations in mice, the suppression of the GABAergic PV+ interneurons caused suppressed gamma oscillations *in vivo*, whereas driving these neurons modulated activity at gamma frequency (Sohal et al., 2009). To summarize, according to the existing findings, the GABA inhibitory system plays a key role in the mechanisms of the generation of gamma oscillations.

Besides the age‐related changes in the strength of 40 Hz ASSR in children, there is another type of auditory response, which changes with age. Several studies have revealed *the decrease* in the amplitude of the transient auditory components (P1, N1, and N2) registered with MEG/EEG (Ceponiene et al., 2002; Ponton et al., 2000; Poulsen et al., 2009; Takeshita et al., 2002). The longitudinal EEG study has demonstrated the larger amplitude of N2 in younger age (10 years) compared to older (11.5 years) in response to frequency‐modulated (40 Hz) tones (Poulsen et al., 2009). The authors hypothesized that one of the possible explanations of this effect can be the drop of synaptic density after 10 years of age. Some studies have also suggested that the GABAergic system regulates synaptic and axonal pruning in developing brain (Gomez‐Castro et al., 2021; Wu et al., 2012) and, probably, a drop of synaptic density in the late childhood may be associated with a relatively late maturation of inhibitory system in the auditory cortex (see Huttenlocher & Dabholkar, 1997). However, it is important to note that the relationship between GABA and auditory Event‐Related Fields (ERF)/Potentials registered with MEG/EEG is hypothetical and it is unknown what exactly the evoked responses reflect in the neural circuitry of the auditory cortex.

In this regard, it may be beneficial to use tones with 40 Hz amplitude modulation for investigating the age‐related changes in auditory responses. It is known that such stimuli (as well as click train) evoke two types of auditory responses in MEG—40 Hz steady‐state gamma response and auditory evoked response called sustained ERF or “sustained field” (see Herdman, 2011; Keceli et al., 2015; Stroganova et al., 2020). If, as we hypothesize, the GABAergic system contributes to the age‐related changes in the strength of both 40 Hz auditory gamma and evoked response (ERF), it is necessary to use a stimulus, which would elicit both types of responses simultaneously. In this case, it would be possible to assess their age‐related changes in the same cortical area at the source level. To the best of our knowledge, there are only two MEG studies in children, in which both types of responses—40 Hz ASSR and sustained ERF—were investigated (Herdman, 2011; Stroganova et al., 2020). However, in these studies, the sources of ASSR and ERF *were different*, so, the authors did not aim to measure the evoked response of the same cortical region as for ASSR and, subsequently, for the same neuronal populations which generated the auditory gamma oscillations.

In the present study, we used MEG to investigate the age‐related changes in the primary auditory cortex, analyzing both 40 Hz ASSR and ERF to the same amplitude‐modulated tones at the source level (in the same region) in 7‐to‐12‐year‐old typically developing children. First, we aimed to estimate the individual sources of 40 Hz ASSR in the left and right hemispheres by calculating the inter‐trial phase consistency (ITPC)—a robust measure of the neural response to periodic auditory signal (Tan et al., 2015)—and to explore the individual variability in the topology of responses. Second, we aimed to assess the relationship between both types of auditory responses (ASSR and ERF) and children's age. We expect the *increase* of 40 Hz ITPC and the *decrease* of the amplitude of ERF with age if the GABA system, as it has been hypothesized, contributes to the age‐related changes in both types of auditory responses. Finally, we predict that 40 Hz ITPC *would be inversely related* to the amplitude of ERF in the same cortical area, so that the higher strength of 40 Hz ASSR will be associated with the lower strength of ERF.

## 2 METHODS

### 2.1 Participants

A total of 30 typically developing children participated in the study (12 girls, age range 7.06–12.03 years, *M*_age_ = 9.10, *SD* = 1.5). All were right‐handed native Russian speakers with no history of neurological, psychiatric or diagnosed language disorders, and with no vision and/or hearing problems. The children's non‐verbal IQ was screened with the Raven's Colored Progressive Matrices (Raven, 2000), and language abilities were measured with the Russian Child Language Assessment Battery (Arutiunian et al., 2022). According to behavioral testing, all children had a normal non‐verbal IQ and language scores (see Table 1 with demographic information).

**TABLE 1:** The demographic information on the participants

Characteristics	Range	Mean	SD
Mean language score	0.87–0.99	0.95	0.03
*Language production*	0.89–0.99	0.96	0.02
*Language comprehension*	0.85–1.00	0.95	0.03
Non‐verbal IQ	23–36	32.4	2.55

### 2.2 Materials and procedure

The stimuli were tones with 40 Hz amplitude modulation. They were generated with in‐home MATLAB code according to the formula:

$$A=\sin\;\left(2{\mathrm{\pi f}}_{c}t\right)\times \left(1+m\times \cos\left(2\pi f_{m}t\right)\right)$$

where *A* is the amplitude, *f*_*c*_ is the carrier frequency, set to 1000, *m* is the modulation depth, set to 1, *f*_*m*_ is the frequency of modulation, set to 40, *t* is the vector of time points for 1 s of stimulus at a sampling rate of 44,100 Hz.

**FIGURE 1:**
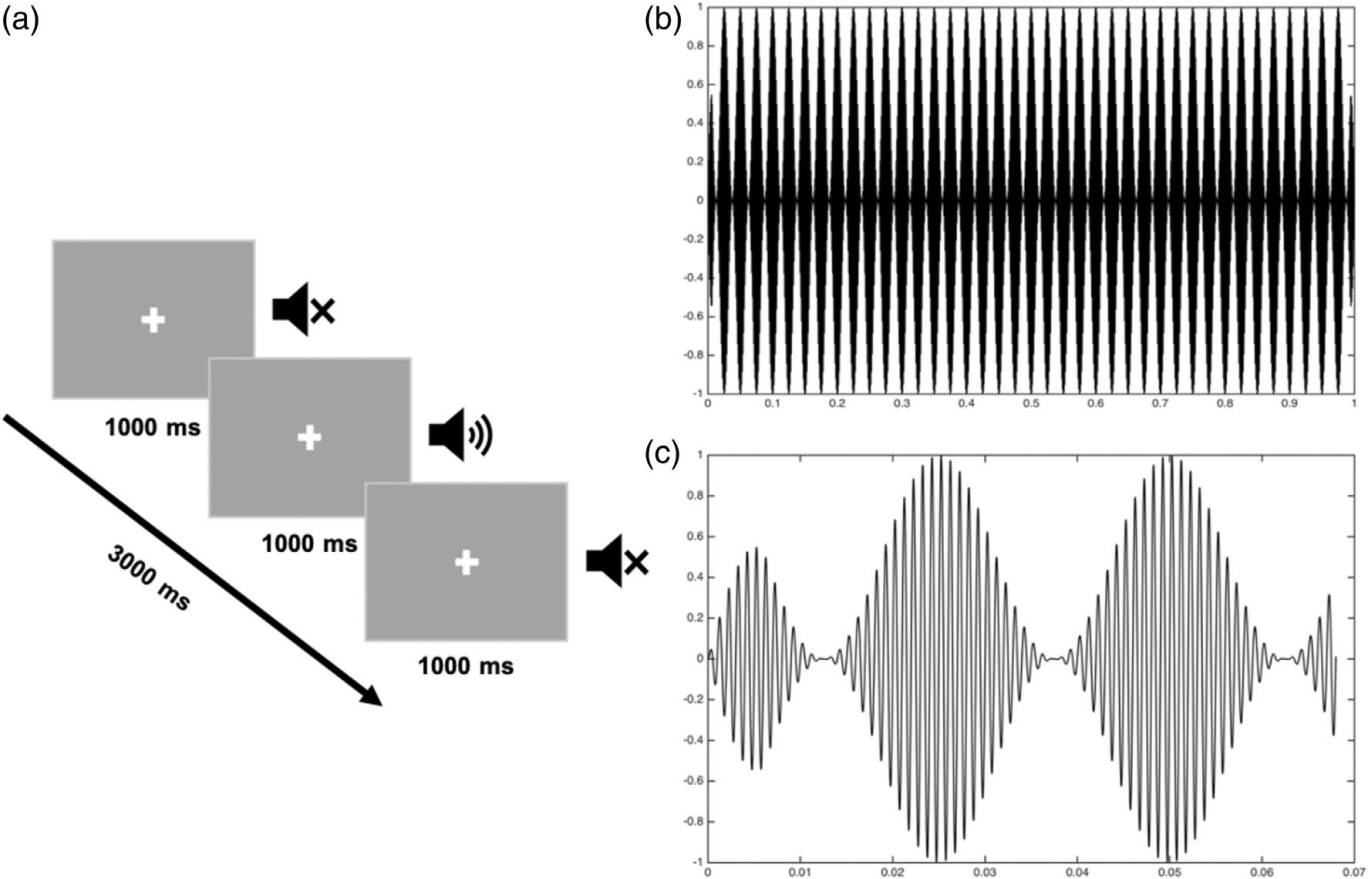
Stimulus presentation: (a) the structure of a trial; (b) pure tone with 40 Hz amplitude modulation (the duration is 1000 ms); (c) the first 70 ms of the amplitude‐modulated tone (for visualization purpose)

The experiment was programmed and run with the PsychoPy software (Peirce, 2007). The presentation of stimuli was binaural with 2000 ms inter‐stimulus interval (Figure 1). Overall, 90 amplitude‐modulated tones (each with 1000 ms duration) were presented during one ~5 min block. Stimuli were delivered via plastic ear tubes with foam tips inserted into the ear canals, and the mean power (intensity) was set at 83.7 dB sound pressure level. In addition to the triggers sent by PsychoPy to the MEG acquisition system, an analog channel of the MEG system recorded the onset of the actual sound sent to the ear tubes. Then, the signal from the analog channel was used to adjust the triggers and, thus, to compensate for the delay between sound presentation by the software and its actual arrival to the ears. In order to reduce eye movements, we asked the children to look at the fixation cross on the screen in front of them during the experiment.

### 2.3 Structural magnetic resonance imaging (MRI)

The high‐resolution whole‐brain structural MRIs of each child were acquired with a 1.5 T Siemens Avanto scanner (repetition time = 1900 ms, echo time = 3.37 ms, flip angle = 15°, matrix size = 256 × 256 × 176, voxel size = 1.0 × 1.0 × 1.0 mm^3^). The MRI segmentation and the reconstruction of the cortical surface from the individual MRIs were performed with the FreeSurfer software (Dale et al., 1999). This surface was then imported to the Brainstorm toolbox (Tadel et al., 2011) and down‐sampled to 15,000 vertices for each participant. Co‐registration between MRI and MEG data was performed with the Brainstorm toolbox based on the six reference points (left and right pre‐auricular points, nasion, anterior and posterior commissure, and interhemispheric point) and the additional digitized head points (*N* = ~150 for each child).

### 2.4 MEG data collection and pre‐processing

MEG was recorded in a sitting position in a magnetically shielded room with a whole‐head 306‐channel MEG (Vectorview, Elekta Neuromag), consisting of 204 orthogonal planar gradiometers and 102 magnetometers. The children's head position within the MEG helmet was tracked every 4 ms during the experiment via four head position indicator (HPI) coils, which were digitized together with fiducial points using 3D digitizer “Fastrak” (Polhemus). The temporal signal space separation method (Taulu & Simola, 2006) and movement compensation procedure implemented in MaxFilter software (Elekta Neuromag) were applied to remove external interference signals, generated outside the brain, as well as to compensate for head movements. An electrooculogram (EOG) was recorded using four electrodes, which were placed above and below the left eye (to detect the blinks) as well as at the left and right outer canthi (to detect horizontal eye movements). Additionally, electrocardiography (ECG) was monitored with ECG electrodes to compensate for cardiac artifacts. The children's heart is located quite close to the MEG helmet, resulting in artifacts from the heartbeat on the MEG data.

MEG was recorded at 1000 Hz sampling rate and filtered off‐line with a band‐pass filter of 0.1–330 Hz for the time‐frequency (TF) analysis and 0.1–45 Hz for the ERF analysis. We applied a notch filter of 50 Hz to reduce powerline noise. Biological artifacts (heartbeats and eye movements) were cleaned with the EEGLAB (Delorme & Makeig, 2004) Independent Component Analysis (ICA), implemented in Brainstorm.

The cleaned MEG data were cut in 3000 ms epochs, ranging from −1500 ms to 1500 ms, and DC offset correction from −100 ms to −2 ms was applied. Epochs were inspected visually and those affected by the muscular artifacts were manually rejected. The minimum number of artifact‐free epochs per subject was 72 (*M*_number_ = 83, *SD* = 4, range 72–87).

### 2.5 MEG source estimation

Only gradiometers were used for the analysis. The individual head models were built with the “Overlapping spheres” method (Huang et al., 1999), which fits one sphere for each sensor. The inverse problem was solved with the depth‐weighted linear L2‐minimum norm estimate method, MNE (Lin et al., 2006), with the dipole orientation constrained to be normal to the cortical surface. The regularization parameter (*λ* = 0.33) was used when computing the inverse operator in order to avoid numerical instability (Hämäläinen & Ilmoniemi, 1994). A common imaging kernel was computed and then applied to obtain single epoch cortical reconstructions. A noise covariance matrix for source estimation was calculated from a 2 min empty room recording, taken after each participant's recording session. To provide comparison between participants, the individual MNEs were projected to the “MRI: ICBM152” template brain provided by Brainstorm.

According to the previous studies (Farahani et al., 2021), cortical generators of ASSR are spread over the auditory regions in both hemispheres. In order to estimate the sources of 40 Hz ASSR, we selected the following regions of interests (ROIs) corresponded to the “core auditory area”: transverse temporal gyrus, transverse temporal sulcus, planum temporale of the superior temporal gyrus, lateral superior temporal gyrus, superior temporal sulcus, planum polar of the superior temporal gyrus, and inferior part of the circular sulcus of the insula in the left and right auditory cortices, according to the Destrieux parcellation cortical atlas (Destrieux et al., 2010).

TF analysis at the source level was performed with the Morlet wavelets (central frequency = 40 Hz, time resolution = 0.3 s). We calculated 40 Hz Inter‐Trial Phase Consistency (ITPC), which computes the phase consistency across the trials, according to the formula:

$$\text{ITPC}=\left|n^{–1}\sum_{r\;=\;1}^{n}e^{ik_{tr}}\right|$$

where *n* is the number of trials, *e* ^*ik*^ is the complex polar representation of phase angel *k* on trial *r*, at the timepoint *t*. The phases were estimated from the Morlet wavelet coefficients. ITPC can take values from 0.0 to 1.0, whereby higher values indicate higher consistency of phases across the trials (Nash‐Kille & Sharma, 2014). TF maps were normalized with an Event‐Related Perturbation (Event‐Related Synchronization/Desynchronization) approach with the baseline period of −500 to −200 ms, evaluating the deviation from the mean over the baseline, according to the formula: (x – mean)/mean  × 100. Such a time window was chosen to avoid the edge effects (Cohen, 2014).

For each child, normalized ITPC‐values were averaged in the 39–41 Hz frequency range in the interval from 200 to 900 ms after stimulus onset (referred to as “40 Hz ITPC”). This time interval was chosen based on the previous studies, which showed that the auditory cortex reaches a steady state after about 200 ms of 40 Hz entrainment (Larsen et al., 2018). In order to reveal the cortical sources of ASSR, we estimated MNI coordinates of 30 vertices with the highest 40 Hz ITPC‐values in defined ROIs (15 vertices per hemisphere), and for the further analysis, extracted 40 Hz ITPC‐values averaged over these 15 vertices in the time interval between 200 and 900 ms in each hemisphere for each child. We have chosen such an approach because it takes into account the individual variability of responses within the defined ROIs and provides more precise source estimation as it has been shown in the previous study (Stroganova et al., 2020).

To provide ERF analysis, the signal was averaged over epochs, and the individual time course was given for each of 15,000 vertices. Then, the cortical map was normalized with a *z*‐score, using the pre‐stimulus time window of −100 to −2 ms. For each child, *z*‐score normalized absolute values were averaged in the time interval between 200 and 1000 ms and were extracted for the individual ROIs as for 40 Hz ASSR (according to MNI coordinates of defined ROIs) in the left and right hemispheres. Such a window of interest for ERF analysis was chosen because the “sustained” component of the evoked response begins at around 200 ms and stays stable during auditory presentation. We applied a special smoothing function based on the Gaussian smoothing (full width at half maximum, FWHM = 3 mm).

### 2.6 Statistical analysis

First, in order to examine the possible age‐related changes in the location of 40 Hz ASSR, we correlated MNI coordinates and children's age in the left and right hemispheres. Second, to estimate the hemispheric differences in 40 Hz ASSR and assess the relationships between two types of auditory responses (ASSR and ERF), we fitted a linear mixed‐effects model, which included the main effect of hemisphere (left vs. right, intercept corresponding to right) and the source amplitude (ERF) nested within the left and right hemispheres separately as fixed effects and the participants as a random intercept. The structure of the model was as follows: lmer(ITPC ~1 + Hemisphere/ERF + (1|ID), data = data, control = lmerControl(optimizer = “bobyqa”)). Finally, we correlated the 40 Hz ITPC and source amplitude with the age for both hemispheres to analyze the association between auditory responses (ASSR and ERF) and children's age.

All models were estimated in R (R Core Team, 2019) with the *lme4* (Bates et al., 2015) and *ggpubr* (Kassambara, 2020) packages. The table for model outcome (Tables 3) was created with the *sjPlot* package (Lüdecke, 2020), and the data were plotted with *ggplot2* (Wickham, 2016).

## 3 RESULTS

### 3.1 Sensor‐level visualization of auditory response

**FIGURE 2:**
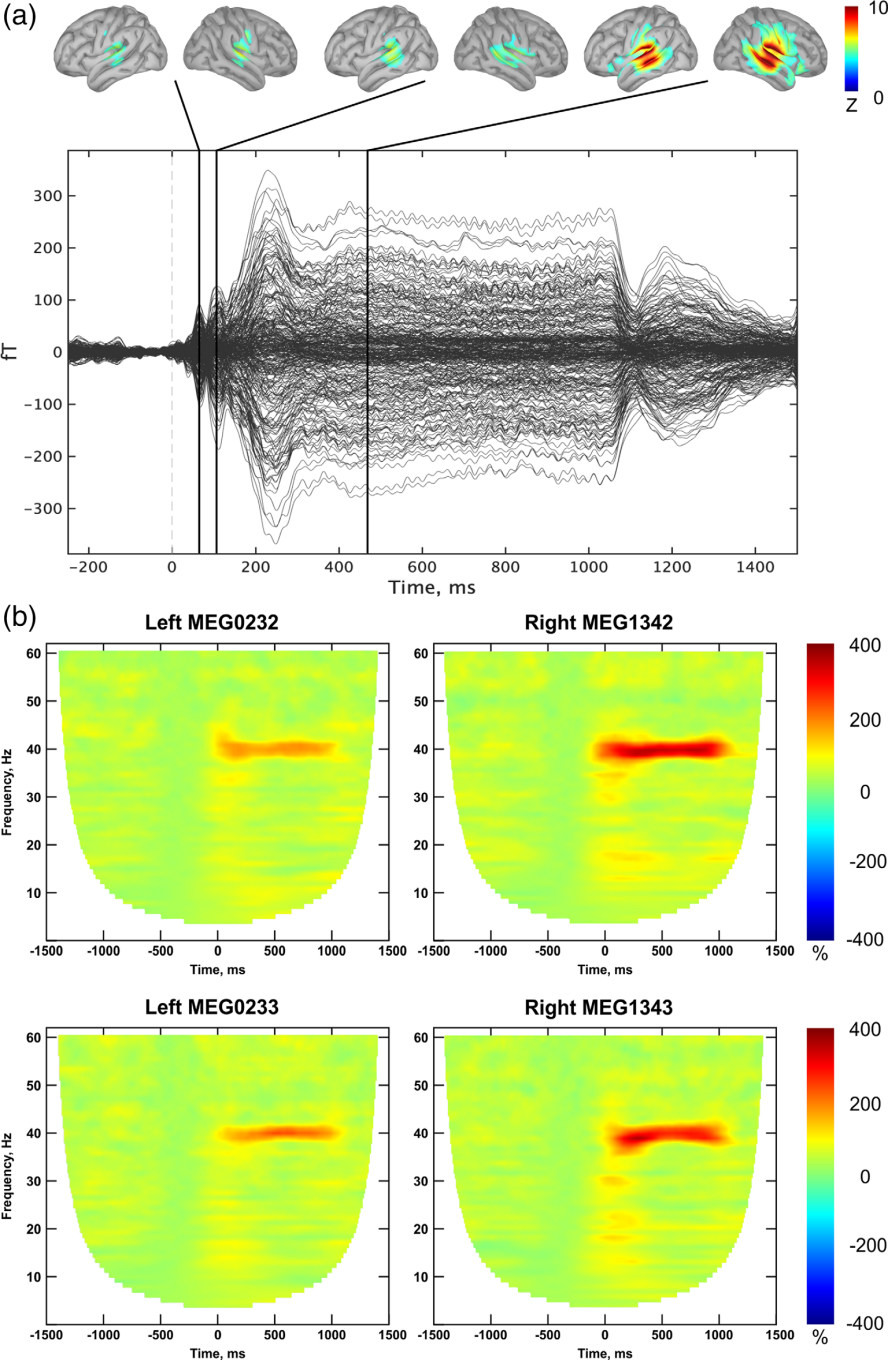
The grand average sensor‐space visualization: (a) “butterfly” plot; the black lines correspond to the first two obligatory transient auditory components (M50 and M100) and sustained part of ERF; the top panel shows the distribution of activity across the cortex at these timepoints; (b) time‐frequency maps of the pairs of gradiometers over the left and right temporal regions with the highest 40 Hz Inter‐Trial Phase Consistency

The visual examination of the raw data demonstrates a clear presence of the first two auditory components (M50 at around 60 ms and M100 at around 110 ms after stimulus onset) and sustained part of ERF (after 200 ms from the stimulus onset) (Figure 2a). The distribution of activity at the source level at these timepoints shows the highest *z*‐score values in the “core auditory area.” Figure 2b also represents the time‐frequency maps of the pairs of gradiometers over the left and right temporal regions with the highest 40 Hz ITPC‐values.

### 3.2 Source estimation of 40 Hz ASSR

In order to reveal the sources of 40 Hz ASSR in the left and right auditory cortices, we calculated the normalized ITPC in the 200–900 ms time window after stimulus onset and estimated MNI coordinates for 15 vertices in each ROI with the highest 40 Hz ITPC‐values. Table 2 represents the grand average MNI coordinates for ASSR in 30 children.

**TABLE 2:** Grand average MNI coordinates of 40 Hz Auditory Steady‐State Response

Left hemisphere			Right hemisphere		
Coordinate	Mean	SD	Coordinate	Mean	SD
X	−49.47	15.56	X	52.46	5.33
Y	−22.25	14.56	Y	−23.85	6.12
Z	6.53	13.73	Z	10.51	6.25

**FIGURE 3:**
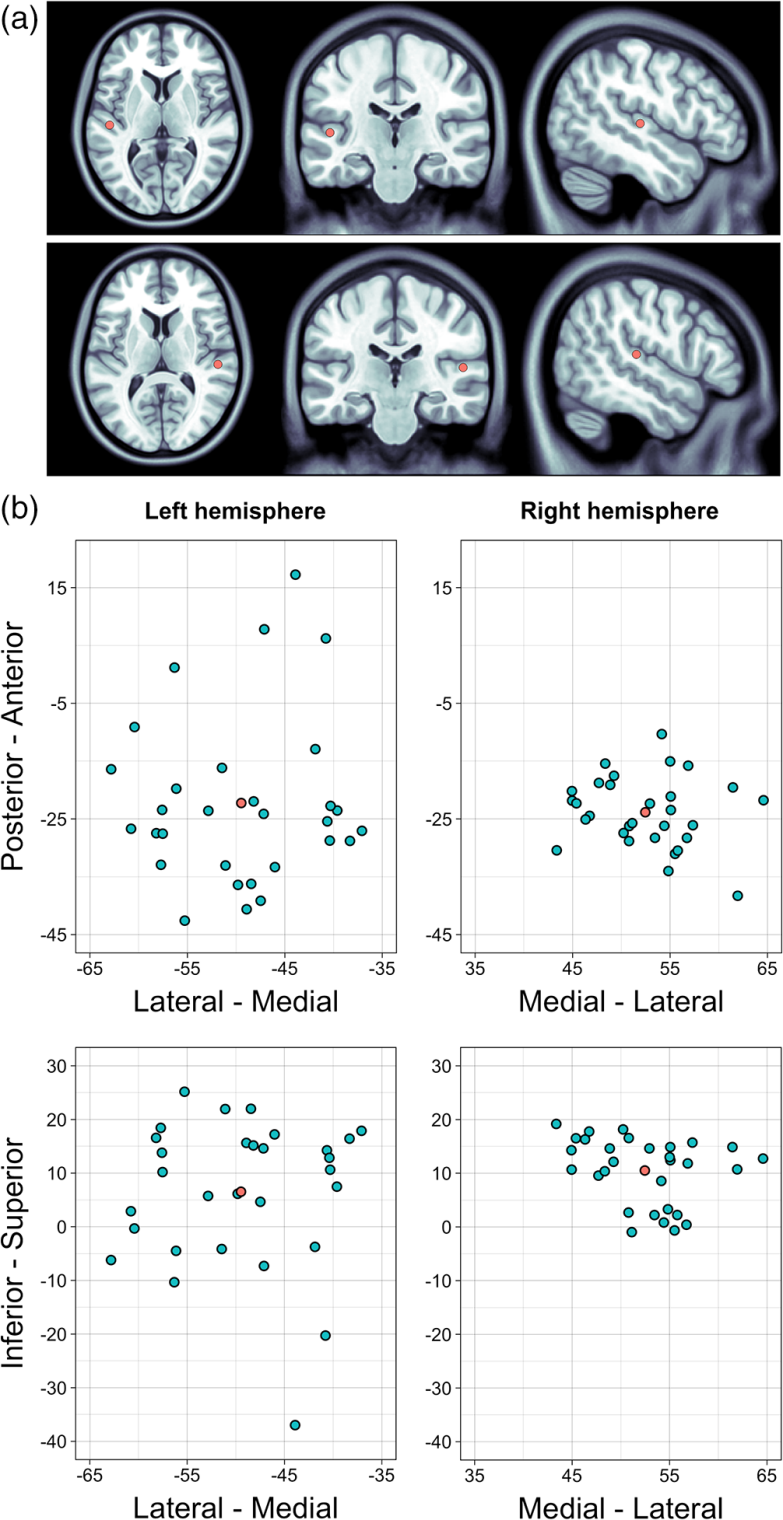
The localization of 40 Hz Auditory Steady‐State Response (ASSR) in MNI space: (a) grand average MNI coordinates of 40 Hz ASSR in axial, coronal, and sagittal directions; (b) MNI coordinates of 40 Hz ASSR in the left and right hemispheres: Blue dots represent the individual MNI coordinates of each child, red dots represent the grand average MNI coordinates.

These results are in line with the previous studies, estimating the sources of 40 Hz ASSR in the left and right auditory cortices (Keceli et al., 2015; Stroganova et al., 2020). Figure 3 visualizes the localization of 40 Hz ASSR in axial, coronal, and sagittal views as well as both individual and grand average MNI coordinates of the response in both hemispheres. The results of source estimation (grand average MNI coordinates) showed that the location of 40 Hz ASSR in the left hemisphere was the *transverse temporal sulcus*, whereas for the right hemisphere it was the *planum temporale of the superior temporal gyrus*.

To examine whether the localization of 40 Hz ASSR changed with age, we used Pearson's correlations (see Section 2.6 for details). We found a significant relationship between children's age and Y coordinate of the MNI space in the right hemisphere, indicating that the topology of 40 Hz ASSR was more posterior in older children, *R* = −.45, Bonferroni‐corrected *p* = .03. Other effects were non‐significant: *left hemisphere*, X, *R* = −.26, *p* = .17; Y, *R* = .08, *p* = .67; Z, *R* = −.14, *p* = .46; *right hemisphere*, X, *R* = .17, *p* = .37, Z, *R* = −.10, *p* = .58.

**FIGURE 4:**
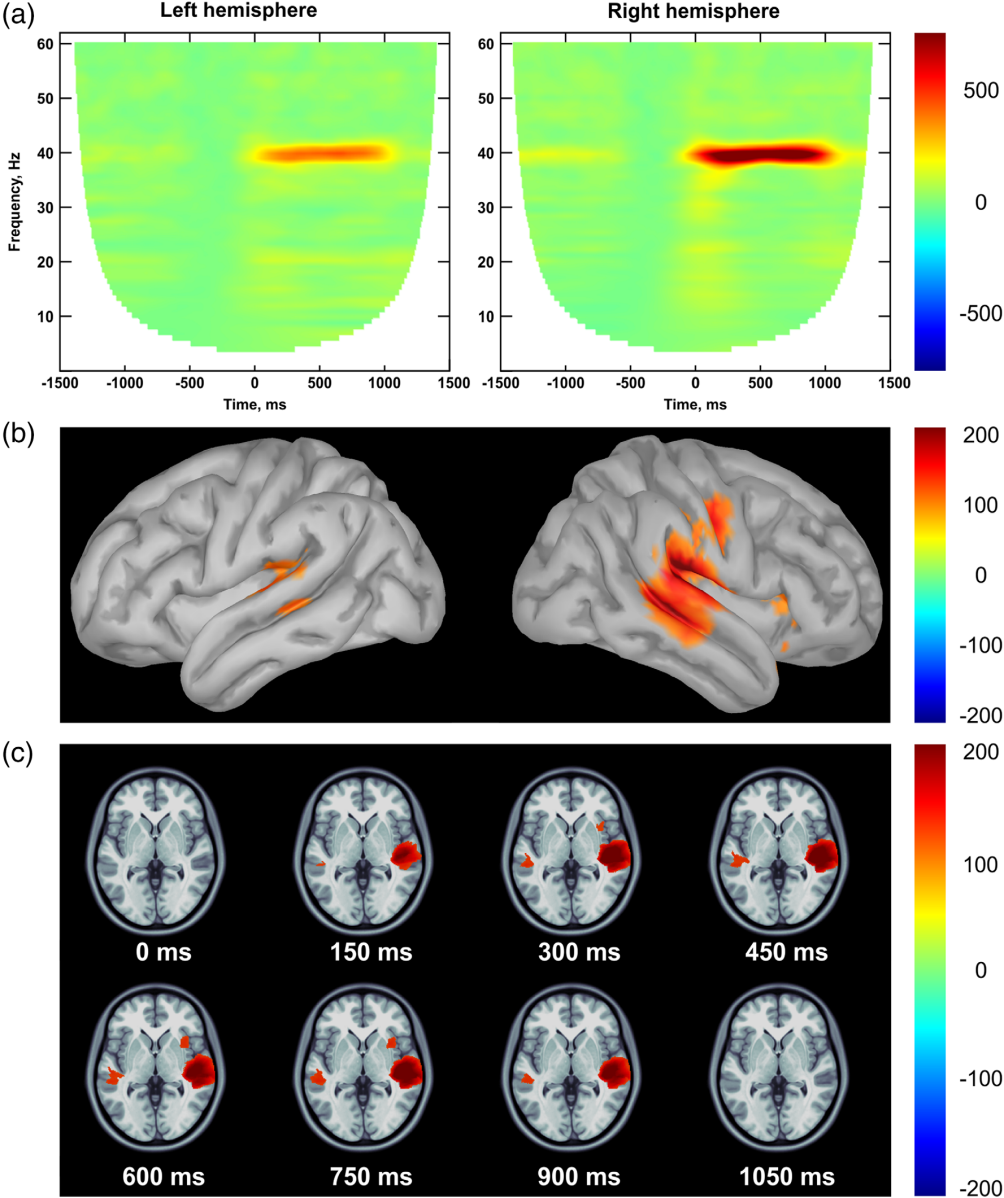
The 40 Hz Auditory Steady‐State Response (ASSR) mapping, grand average: (a) time‐frequency maps for the left and right auditory regions of interests (ROIs) (normalized Inter‐Trial Phase Consistency, % change from the baseline); (b) distribution of 40 Hz ASSR across the cortex in the left and right hemispheres averaged in a 200–900 ms time interval; (c) 40 Hz steady‐state gamma synchronization during auditory stimulation (0–1050 ms); neurological convention. The amplitude threshold is set to 70% of the highest values for graphical purposes in (b) and (c).

Figure 4 shows the grand average time‐frequency maps (normalized 40 Hz ITPC) for the left and right auditory ROIs as well as cortical distribution of 40 Hz ASSR, averaged in time (200–900 ms), and 40 Hz gamma synchronization during auditory stimulation.

**FIGURE 5:**
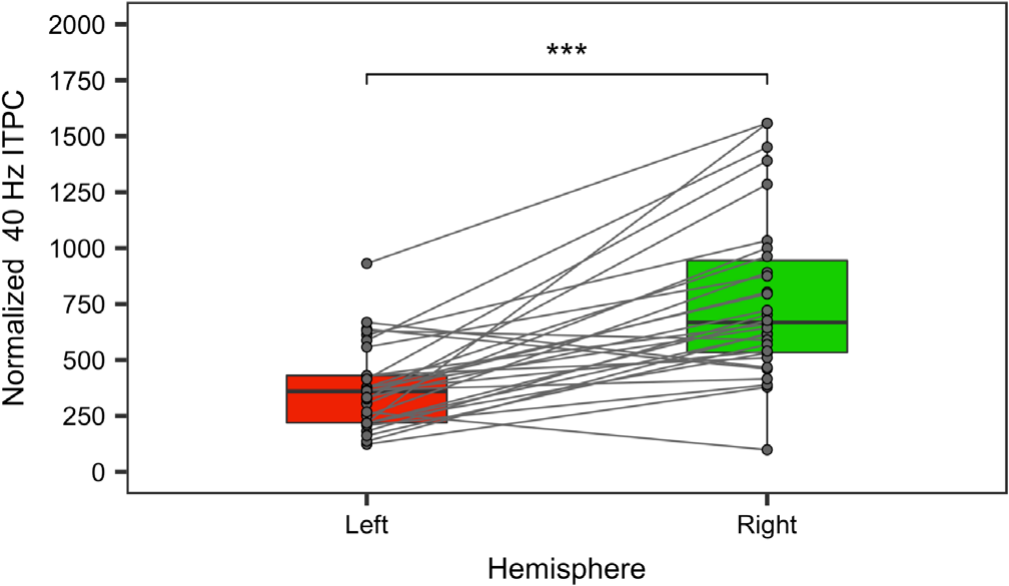
Normalized 40 Hz intertrial phase consistency (ITPC) in the left and right auditory regions of interests (ROIs). The dots represent the individual results of each child.

The results revealed a significant right hemispheric dominance of 40 Hz ASSR, indicating higher ITPC‐value in the right hemisphere compared to the left, *M*_Left_ = 375.57 (*SD* = 189.12) versus *M*_Right_ = 772.42 (*SD* = 368.98), *β* = −500.99, SE = 79.41, *t* = −5.31, *p* < .001 (Figure 5, Table 3). This is in line with the previous studies in both children (Edgar et al., 2016; Stroganova et al., 2020) and adults (Pellegrino et al., 2019).

**TABLE 3:** The relationships between 40 Hz Auditory Steady‐State Response and the amplitude of sustained part of Event‐Related Fields in the left and right auditory ROIs

	40 Hz Inter‐Trial Phase Consistency			
	Estimate	Standard error	*t*	*p*
(Intercept)	766.28	82.06	9.34	<.001***
Hemisphere_Left	−500.99	79.41	−6.31	<.001***
Hemisphere_Left:Amplitude	12.85	7.50	1.71	.092
Hemisphere_Right:Amplitude	−43.97	11.71	−3.75	<.001***
Random effects				
*σ* ^2^	50,232.26			
*τ*_00ID_	18,112.47			
ICC	0.27			
N_ID_	30			
Observations	60			
Marginal *R* ^2^/Conditional *R* ^2^	0.461/0.604			

*** The sigificance is provided. For random effects, the model does not calculate the *p*‐values

### 3.3 The relationships between 40 Hz ITPC and the amplitude of ERF

**FIGURE 6:**
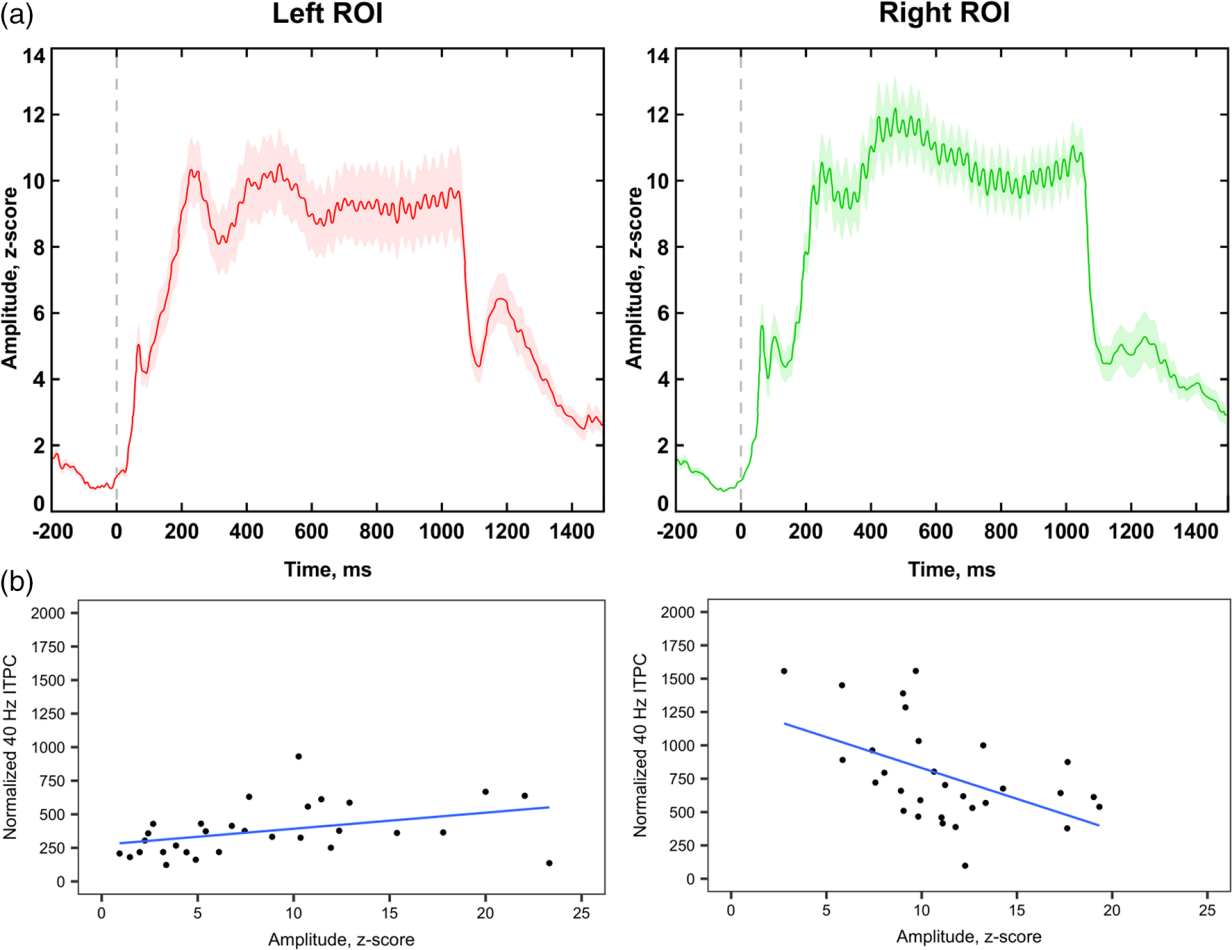
The relationship between 40 Hz Auditory Steady‐State Response (ASSR) and the amplitude of the sustained part of Event‐Related Fields: (a) grand average time‐courses of the estimated sources in the left and right auditory ROIs (amplitude, *z*‐score). The shaded area corresponds to standard error of the mean; (b) the association between 40 Hz Inter‐Trial Phase Consistency (ITPC) and the source amplitude in the left and right hemispheres

A significant association between 40 Hz ASSR and source amplitude was found in the right auditory region, but not in the left: left ROI: *β* = 12.85, SE = 7.50, *t* = 1.71, *p* = .092; right ROI: *β* = −43.97, SE = 11.71, *t* = −3.75, *p* < .001 (Figure 6, Table 3). The results revealed that the higher 40 Hz ITPC was associated with lower source amplitude.

### 3.4 The associations between auditory responses and children's age

**FIGURE 7:**
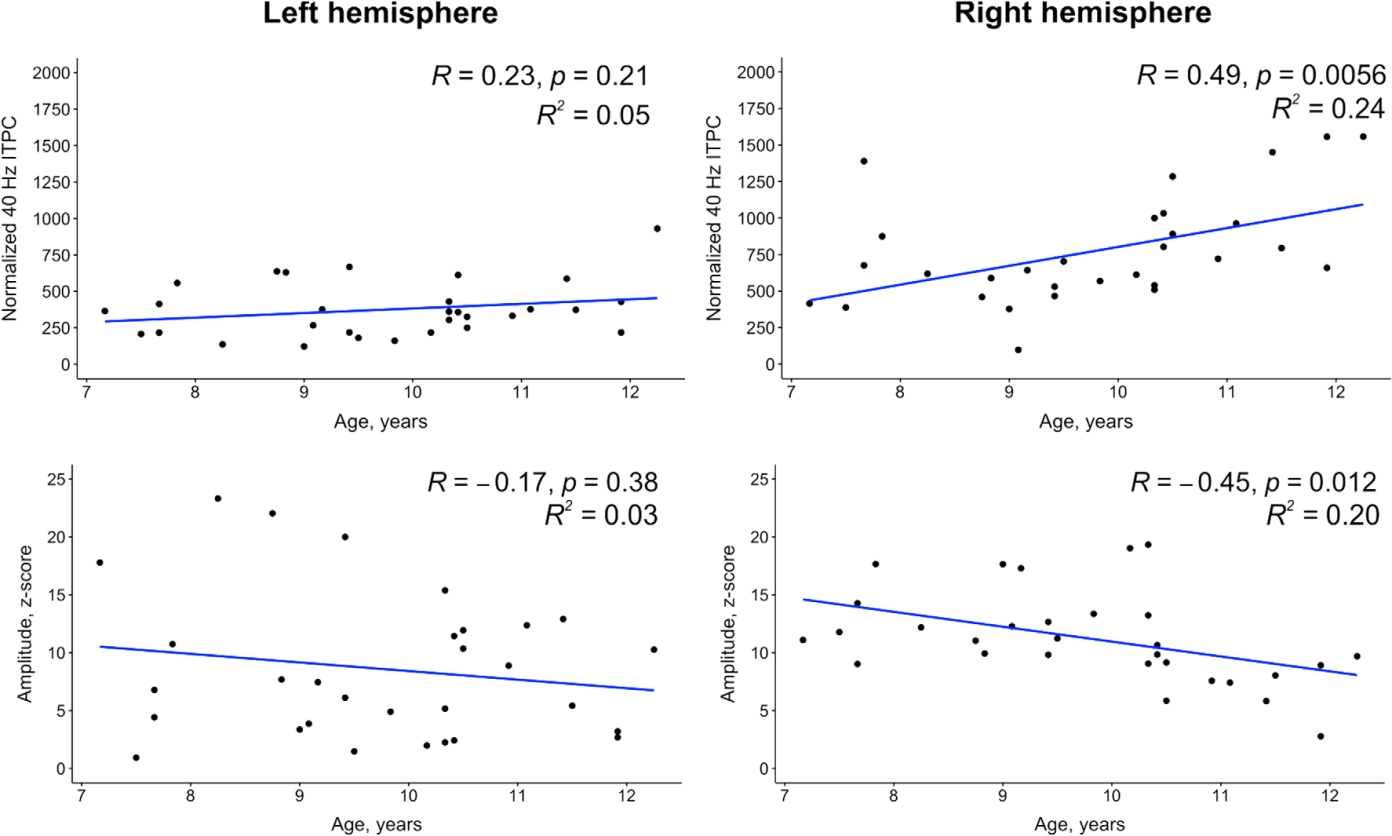
The association between children's age and auditory responses (40 Hz Inter‐Trial Phase Consistency and the amplitude of Event‐Related Field) in the left and right auditory regions of interests (ROIs)

In order to investigate whether there are age‐related changes in both types of auditory responses (40 Hz ASSR and ERF), we calculated Person's correlations between (1) normalized 40 Hz ITPC and children's age and (2) the amplitude of ERF and children's age for the left and right auditory ROIs. The results showed an age‐related *increase* of 40 Hz ITPC, but the effect was significant only for the right hemisphere, left ROI: *R* = .23, *p* = .21; right ROI: *R* = .49, *p* = .005 (Figure 7). We also found a significant relationship in the right hemisphere between the amplitude of ERF and children's age, indicating an age‐related *decrease* of source strength, left ROI: *R* = −.17, *p* = .38; right ROI: *R* = −.45, *p* = .012.

Summarizing, the age‐related changes in both auditory responses as well as in their topology were observed only in the right hemisphere.

## 4 DISCUSSION

The present study addressed the age‐related changes of 40 Hz steady‐state gamma response and ERF in the primary auditory cortex (or in the vicinity of this area) and their relationships to each other in typically developing 7‐to‐12‐year‐old children, using MEG. In general, we found that both source topology (MNI coordinates) and the strength of responses (40 Hz ITPC and the amplitude of ERF) change with age in the right hemisphere.

First, we estimated the sources of 40 Hz ASSR and, in accordance with the previous studies in both children (Stroganova et al., 2020) and adults (Keceli et al., 2015), its localization was in the primary auditory cortex (area A1) or in the adjacent to A1 region, that is, transverse temporal sulcus in the left hemisphere and planum temporale of the superior temporal gyrus in the right hemisphere. Our results demonstrated that individual variability in the topology of 40 Hz ASSR can be partly associated with children's age. We observed the age‐related changes of MNI coordinates of this neural response in the right hemisphere, indicating that the topology of 40 Hz ASSR was more posterior in older children. Some studies have also showed that the auditory responses can change their location with age (e.g., P300 in the study by Tsolaki et al., 2015) but, to the best of our knowledge, our study is the first that revealed the topographical age‐related changes of 40 Hz steady‐state gamma response.

The analysis of phase consistency across the trials (or ITPC)—a robust measure of the neural response to periodic auditory signal (Tan et al., 2015)—revealed a right hemispheric dominance of 40 Hz ASSR, indicating that the ITPC‐value was higher in the right auditory ROI. This is in line with the previous studies in both children (Edgar et al., 2016; Poulsen et al., 2009; Stroganova et al., 2020) and adults (Pellegrino et al., 2019; Ross et al., 2005). Some authors hypothesized that this asymmetry can be explained by the fact that the right auditory cortex is specialized for processing the temporal periodicity of the sound (Ross et al., 2005). The previous MEG studies, investigating the transient auditory components (e.g., P50m, N100m) have also shown a right‐hemispheric dominance of responses (Kohl et al., 2022; Kotecha et al., 2009; Orekhova et al., 2013; Parviainen et al., 2019). Overall, the hemispheric asymmetry of auditory responses can be associated with both morphological and functional differences between left and right auditory cortices (Boemio et al., 2005; Devlin et al., 2003; Hine & Debener, 2007).

In accordance with the previous findings (Edgar et al., 2016; Maurizi et al., 1990; Poulsen et al., 2009; Stapells et al., 1988; Stroganova et al., 2020), we showed a relationship between the strength of 40 Hz ASSR and children's age. Specifically, the results revealed the age‐related increase of 40 Hz ITPC in the right hemisphere in our sample of 7‐to‐12‐year‐old children, and this corresponds to studies which showed a monotonic enhancement of both power and ITPC of auditory gamma oscillations from childhood to the early adolescence (Edgar et al., 2016; Poulsen et al., 2009). It is important to note that studies, using MEG source estimation of 40 Hz ASSR (e.g., Ono et al., 2020; Stroganova et al., 2020), also did not find a significant relationship between the age and 40 Hz ITPC in the left hemisphere (see, however, Edgar et al., 2016). Apparently, the age‐related increase of 40 Hz ITPC can be associated with the maturation of GABAergic inhibitory neurotransmission and developmental changes of E/I balance (Cho et al., 2015). Of note, the increase in the strength of gamma oscillations with age is not unique for the auditory cortex and occurs also, for example, in the visual cortex (Orekhova et al., 2018). In general, this development starts very early in the neonate brain by switching from a depolarizing to hyperpolarizing action of GABA receptors, that is, excitatory‐to‐inhibitory shift of GABA receptors (Ben‐Ari, 2002, 2014; Cherubini et al., 1991).

We also revealed a significant relationship between the amplitude of the evoked response and children's age. Contrary to the age‐related *increase* of 40 Hz ITPC, our findings showed the age‐related *decrease* of the ERF in the right auditory ROI. Although this study is the first that demonstrated the decline in the amplitude of the sustained part of ERF, there are several previous works that indicated the age‐associated decrease in amplitude of the transient auditory components (P1, N1, and N2) in children, using MEG/EEG (e.g., Ceponiene et al., 2002; Ponton et al., 2000; Poulsen et al., 2009; Takeshita et al., 2002). For example, in the only EEG longitudinal study of Poulsen et al. (2009), it has been shown the larger amplitude of N2 in younger age (10 years) compared to older (11.5 years) in response to frequency‐modulated (40 Hz) tones, reflecting the maturational changes in the auditory cortex. One of the potential explanations of the decline in amplitude can be the drop of synaptic density after 10 years of age and an advance of intracortical myelination (see Ponton et al., 2000; Poulsen et al., 2009). An alternative hypothesis, regarding the decrease in N2 amplitude, is related to the development of frontal inhibitory control owing to the known association between N2 and the selective attention effect (Ponton et al., 2000). The results of our study contribute to the previous findings and demonstrate that not only the evoked transient auditory components but also the sustained part of ERF changes its amplitude with age.

Importantly, our study revealed the relationship between two types of neural responses to the same amplitude‐modulated tones (40 Hz steady‐state gamma and ERF), indicating that the higher 40 Hz ITPC was associated with the lower amplitude of ERF in the right hemisphere. As we measured the strength of both auditory responses in the same cortical region and, perhaps, the same neuronal populations generated 40 Hz steady‐state gamma response and ERF, we hypothesized that the age‐related changes in these auditory responses may be associated with the same mechanism. The possible explanation of these age‐associated changes in 40 Hz ITPC and the amplitude of ERF (and, subsequently, the opposite relation of these auditory responses to each other) can be the development of GABAergic inhibitory neurotransmission with age. Some studies have shown that inhibitory interneurons are widely presented in the primary auditory cortex (Studer & Barkat, 2022), mature relatively late in development (Gao et al., 1999), and play a significant role in the generation of 40 Hz steady‐state gamma response (Sivarao et al., 2016). Therefore, GABA maturation contributes to the age‐related increase of 40 Hz ITPC/power (see Edgar et al., 2016), stabilizing the E/I balance. At the same time, GABA regulates synapse elimination and axonal pruning in developing brain (Gomez‐Castro et al., 2021; Wu et al., 2012) and, perhaps, a relatively late maturation of inhibitory system in the auditory cortex can explain a drop of synaptic density in the late childhood (see Huttenlocher & Dabholkar, 1997). Note that the synaptic reduction was used as one of the possible explanations of the decline in amplitude of the transient auditory evoked potentials in children (see Ponton et al., 2000; Poulsen et al., 2009). Taking everything into account, we hypothesize that the development of the GABAergic inhibitory system contributes to both type of auditory responses: 40 Hz ITPC increases with age, whereas the amplitude of ERF decreases with age, and, as a result, these neural responses are oppositely related to each other.

## 5 LIMITATIONS AND FUTURE DIRECTIONS

The study has some limitations, which should be highlighted. First, we did not include the control group of adults; therefore, it is unclear whether the relationship between 40 Hz steady‐state gamma response and the amplitude of ERF would differ in adults from children. Future studies would benefit from including adults into the research because the knowledge of how these auditory responses are related to each other in the group of adults can shed light on the age‐related changes of these responses in children. Second, our sample of participants included 30 children; however, given the large age range of participants, it is needed to include a larger group of children into the study. Moreover, although we showed the age‐related changes in both topology and the strength of the auditory responses, it would be beneficial to examine these changes longitudinally in the same group of children and, thus, understand the maturational effect. Finally, we explained our findings in the light of the GABA system maturation and the development of E/I balance, but we did not measure GABA level directly. In future studies, it is necessary to take into account this limitation and measure GABA level in order to test directly how GABA development predicts the changes in both 40 Hz ASSR and the amplitude of ERF.

## 6 METHODOLOGICAL CONSIDERATIONS ON SOURCE ESTIMATION

It is important to notice that, probably, MEG source estimation technique did not account for the localization of ASSR because MNI coordinates of 40 Hz ITPC highest values in our study corresponded to those reported previously and estimated with sLORETA method (Stroganova et al., 2020) and single dipole modeling (Keceli et al., 2015). However, although the grand average MNI coordinates of 40 Hz ASSR in our study were in line with the previous findings, there was a variability in source locations at individual level. Visual exploration of the individual sources of 40 Hz ASSR (Figure 3b) clearly demonstrated the topographic distribution of neural response across the “core auditory area,” especially in the left hemisphere. Perhaps, this variability can be explained by both age‐related maturational changes in the auditory cortex, which start very early in development and continue until early adolescence (Shaw et al., 2008) and also by the interindividual differences with respect to the shape and structural properties of the area A1 (Zoellner et al., 2019).

Contrary to Edgar and colleagues' MEG study (Edgar et al., 2016) with the similar age‐range group, we detected a clear 40 Hz ASSR in all children from our sample, whereas in Edgar et al. (2016) study, 40 Hz ASSR was not observed in the majority of children. Importantly, in a more recent study (Stroganova et al., 2020), 40 Hz ASSR has also been detected in most of the children. Stroganova et al. (2020) speculated that, in comparison to Edgar et al. (2016) study, they could detect a clear steady‐state 40 Hz gamma response in most children owing to methodological differences, such as, for example, the type of the stimuli (clicks instead of the amplitude‐modulated tones), stimulus presentation (monaural instead of binaural), and source localization methods (sLORETA instead of single dipole modeling). However, because we used the same paradigm as in Edgar et al. (2016) study with amplitude‐modulated tones presented binaurally, we suppose that we could detect 40 Hz ASSR because we applied another source estimation technique. Farahani et al. (2021) showed that there is individual variability in the localization of 40 Hz ASSR even in adults. This suggests that to estimate precisely the sources of 40 Hz steady‐state gamma response, we need to take into account multiple anatomical ROIs in the “core auditory area” (see Section 2) to explore inter‐individual differences across children. Moreover, our findings demonstrated that children's age may account for the topology of 40 Hz ASSR and may be one of the possible factors contributing to the variability in response location.

## 7 CONCLUSIONS

To conclude, the present study provided some new evidence on the age‐related changes in the topology of 40 Hz ASSR and in the strength of both types of auditory responses (steady‐state 40 Hz gamma and ERF). Numerous studies have revealed that the abnormalities in gamma‐band oscillations could be the potential neural markers in such neuropsychiatric disorder as autism (Gandal et al., 2010; Levin & Nelson, 2015; Rojas & Wilson, 2014; Rubenstein & Merzenich, 2003; Subramanian et al., 2018). Therefore, our findings in the group of 7‐to‐12‐year‐old typically developing children may potentially contribute to further clinical research.

## AUTHOR CONTRIBUTIONS

**Vardan Arutiunian**: Conceptualization. **Vardan Arutiunian, Giorgio Arcara**: Methodology. **Vardan Arutiunian, Irina Buyanova, Militina Gomozova**: Investigation. **Vardan Arutiunian, Militina Gomozova**: Data curation. **Vardan Arutiunian, Giorgio Arcara**: Formal analysis. **Vardan Arutiunian:** Writing – Original Draft. **Vardan Arutiunian, Giorgio Arcara, Olga Dragoy:** Writing – Review and Editing. **Vardan Arutiunian**: Project administration. **Olga Dragoy**: Resources. All authors read and approved the final manuscript.

## CONFLICT OF INTEREST

The authors have no conflict of interest to report.

## Data Availability

The datasets of the current study are available from the corresponding author upon reasonable request. The Matlab code for the generation of amplitude‐modulated tones is freely available online: https://github.com/giorgioarcara/MEG-Lab-SC-code/tree/master/tDCS-ASSR. The R codes for statistical analysis, plotting, and building table with model outcome are freely available online: https://osf.io/2ga6h/.
